# Completeness and Changes in Registered Data and Reporting Bias of Randomized Controlled Trials in ICMJE Journals after Trial Registration Policy

**DOI:** 10.1371/journal.pone.0025258

**Published:** 2011-09-21

**Authors:** Mirjana Huić, Matko Marušić, Ana Marušić

**Affiliations:** 1 Agency for Quality and Accreditation in Health Care, Zagreb, Croatia; 2 Department of Research in Biomedicine and Health, University of Split School of Medicine, Split, Croatia; 3 Croatian Centre for Global Health, University of Split School of Medicine, Split, Croatia; Medical Research Council South Africa, South Africa

## Abstract

**Objective:**

We assessed the adequacy of randomized controlled trial (RCT) registration, changes to registration data and reporting completeness for articles in ICMJE journals during 2.5 years after registration requirement policy.

**Methods:**

For a set of 149 reports of 152 RCTs with ClinicalTrials.gov registration number, published from September 2005 to April 2008, we evaluated the completeness of 9 items from WHO 20-item Minimum Data Set relevant for assessing trial quality. We also assessed changes to the registration elements at the *Archive* site of ClinicalTrials.gov and compared published and registry data.

**Results:**

RCTs were mostly registered before 13 September 2005 deadline (n = 101, 66.4%); 118 (77.6%) started recruitment before and 31 (20.4%) after registration. At the time of registration, 152 RCTs had a total of 224 missing registry fields, most commonly ‘Key secondary outcomes’ (44.1% RCTs) and ‘Primary outcome’ (38.8%). More RCTs with post-registration recruitment had missing Minimum Data Set items than RCTs with pre-registration recruitment: 57/118 (48.3%) vs. 24/31 (77.4%) (χ^2^_1_ = 7.255, *P* = 0.007). Major changes in the data entries were found for 31 (25.2%) RCTs. The number of RCTs with differences between registered and published data ranged from 21 (13.8%) for Study type to 118 (77.6%) for Target sample size.

**Conclusions:**

ICMJE journals published RCTs with proper registration but the registration data were often not adequate, underwent substantial changes in the registry over time and differed in registered and published data. Editors need to establish quality control procedures in the journals so that they continue to contribute to the increased transparency of clinical trials.

## Introduction

Study publication and outcome reporting biases are two major obstacles to evidence-based practice because they overestimate the effect of experimental treatments, can cause harm and are unethical [1]–[18]. In recent years, the transparency of clinical trials has considerably increased with the establishment of public clinical trial registries and legislative changes in many countries [19]–[22]. An important momentum for increased transparency in research and reporting in medicine was provided by the International Committee of Medical Journals' (ICMJE) in 2004, when it put forward its policy on mandatory registration of clinical trials as a precondition for manuscript submission [23]. The ICMJE also contributed to the development and adopted the first version of 20-item World Health Organization (WHO) Minimum Data Set in 2005, making clear that the journals “will consider a registration data set inadequate if it has missing fields or fields that contain uninformative terminology” [24]. In February 2006, Item 11 ‘Research Ethics Review’ from the 20-item WHO Minimum Data Set was changed to ‘Countries of recruitment’[25]. In 2007, ICMJE expanded the acceptable primary registers to all those participating in the WHO International Clinical Trials Registry Platform (ICTRP) and implemented the WHO definition of a clinical trial [21].

The initial ICMJE registration policy in 2004 set two deadlines for trial registration [23]. The first deadline was 1 July 2005 – for any clinical trial starting enrollment after that date, they had to be registered with the full Minimum Data Set entered before the recruitment of the first patients. The second deadline was 13 September 2005, by which time all trials that began enrollment prior to 1 July 2005, had to be registered in order to be considered for publication after manuscript submission.

Both the scientific community and trial funders embraced the policy and the experience of the largest trial registry, ClinicalTrials.gov, showed that the number of registered trials increased dramatically around the second ICMJE deadline and kept a steady increase since then [26]. Despite increased registration, the completeness and quality of registered data and the reporting bias still remain as an obstacle to trial transparency [27].

In a cross-sectional study of trials in ClinicalTrials.gov registry, Ross et al [28] demonstrated that, while the registration of mandatory registry data was satisfactory, the reporting of optional data for ClinicalTrials.gov but mandatory data for ICMJE, such as primary outcome and trial end date, was unsatisfactory. In a study looking at randomized controlled trials (RCTs) published in 10 high-impact general and specialty medical journals [29] selective outcome reporting was still prevalent, despite the registration of the trials in public registries.

As these studies were performed on a general population of registered trials and journals, it is possible that their results reflect the fact that other journals may have not fully adopted the ICMJE registration policy. To assess how well ICMJE member journals followed their own registration requirement policy in the first two years of the policy implementation [23], [24] we evaluated the completeness of 9 out of 20 WHO Minimum Data Set items, which are relevant for assessing trial quality. We analyzed articles reporting on RCTs in ICMJE member journals from the 13 September 2005 deadline to April 2008, registered in the largest public trial registry at that time, ClinicalTrials.gov. As ClinicalTrials.gov also registers all changes to RCTs records in its *Archive* site, we evaluated the history of changes to the registration data elements until the date of publication. Finally, we assessed the reporting bias by determining the differences in the published vs. registered data.

## Methods

### Data sources and study period

We retrospectively identified all reports of clinical trials with ClinicalTrials.gov registry number (NCT), published by ICMJE journals from 13 September 2005 to 24 April 2008 (total time: 2.5 years after the second ICMJE deadline on 13 September 2005). Reports were identified by a PubMed search using ‘ClinicalTrials.gov [si]’ as a key word and the following limits: all individual ICMJE journals, date range (YYYY/MM/DD) and type of article (RCT).

ClinicalTrials.gov database was chosen for analysis because it was one of the largest public registry of clinical trials at the time of the first ICMJE registration policy and because it maintains an *Archive* feature, which records all modifications to individual trial database records. The *Archive* site was implemented on 23 June 2005 and was open to the public from 6 October 2006 at http://ClinicalTrials.gov/archive [30].

### Sample

**Figure 1:**
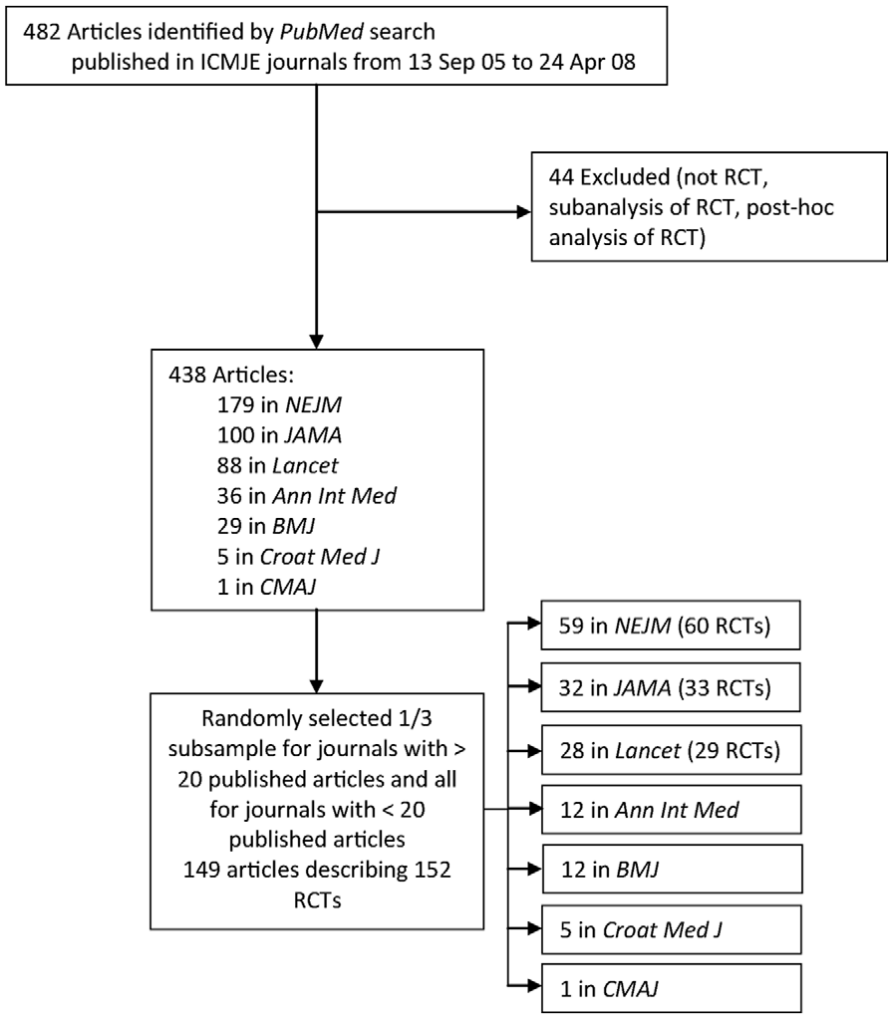
Flow diagram of RCTs sample selection from all reports of clinical trials with ClinicalTrials.gov registration number published by ICMJE journals from 13 September 2005 to 24 April 24 2008.

The criteria for including articles in the study were: 1) presenting results of RCTs and published in an ICMJE journal from 13 September 2005 to 24 April 2008, 2) having ClinicalTrials.gov registration number, and 3) having visible registration data in the *Archive* site of ClinicalTrials.gov (after 23 June 2005). From 482 retrieved articles, we excluded non-RCTs articles and articles describing sub-analyses or post hoc analyses of RCTs (total n = 44). This left 438 articles from 5 large ICMJE journals with >20 published articles and 2 journals with <20 published articles; other ICMJE journals did not published articles about RCTs in the studied period. For journals with smaller volume of published articles, we included all of them (5 in *Croatian Medical Journal*, *Croat Med J*, and 1 in *Canadian Medical Association Journal*, *CMAJ*) and took a random third from each of the other ICMJE journals with more than 20 published articles (Figure 1). The randomization was performed using random permutation of integers (available at www.randomization.com).

### Data extraction

#### Nine WHO Minimum Data Set items

One investigator extracted from ClinicalTrials.gov the data for 9 out of 20 WHO Minimum Data Set items [31]: Item 10 (‘Official scientific title of the study – Scientific Title’), Item 12 (‘Health Condition(s) or Problem(s) Studied’), Item 13 (Interventions), Item 14 (‘Key a) inclusion or b) exclusion criteria’), Item 15 (‘Study type’), Item 16 (‘Anticipated trial start date – Date of First Enrollment’), Item 17 (‘Target sample size’), Item 19 (‘Primary outcome’) and Item 20 (‘Key secondary outcomes’). Data for Minimum Data Set items 1, 2, 3, 6, 7, 8, 9 and 18 were not assessed because they are either administrative in character, expected to differ at first and last registration, were recorded only in the recruiting period or were not routinely published in a journal article. The definitions of the WHO Minimum Data Set were taken from the WHO Trial Registration Data Set (Table S1) [31]. The other investigator verified the extraction and data entries and disagreements were resolved by discussion. The following parameters were recorded: 1) data at first registration, 2) data at last registration modification before publication, and 3) differences of published data to those specified in the registration entry. As all journals did not provide the date of manuscript submission, the date of publication was used as the proxy for the last date of registration entry change before article submission to a journal.

### Completeness of Minimum Data Set items in the register

For data set items included in the study we identified those with: a) missing information – defined as the data not visible in the specified registry item, or b) with uninformative terminology – defined as unspecified or unclear information for the relevant registry item (e.g., a code instead of a generic name of drug for Item 13), both at the time of the first registration and the time of the last registration modification before publication.

### Changes in the Minimum Data Set Items in the registry

We also identified Minimum Data Set items for which the information was changed from the first to the last registration modification prior to publication, as well as from the last modification prior to publication and the published article. A major modification was defined as: a) qualitative change – the difference in the meaning of the information provided in a registry field (e.g., addition or deletion of data items in the article vs. that in the registry, direction of change in time, uninformative entries), or b) quantitative change – difference in a numerical entry in a registry field. Minor changes, such as administrative or grammatical changes, were also recorded but were not included in the analysis. Trials with a single registration point in the same register were defined as those that had no recorded changes in the registry.

### Reporting completeness of Minimum Data Set items

The definition of major modifications was the same as used for modifications in the registry, except for primary and secondary outcomes, for which we used a modified classification of Chan et al. [8]: 1) primary and secondary outcome not visible in the specified registry item; 2) registered primary outcome reported as a secondary outcome in the published article; 3) registered primary and secondary outcome omitted in the published article; 4) a new primary and secondary outcome introduced in the published article; 5) difference in the timing of assessment of the registered and published primary and secondary outcome; and 6) the outcome used in the power calculation not the same in the register and the published article.

### Statistical analysis

The data were presented as frequencies and percentages or median with 95% confidence intervals (CI). Chi-square test was used to compare missing registry data. To ensure that the random selection of one third of the published articles did not favor any particular period after the September 2005 deadline, possible differences in the number of articles published in three separate periods (Sept 2005 to Apr 2006, May 2006 to Apr 2007 and May 2007 to Apr 2008) were tested.

## Results

### Characteristics of RCTs from ClinicalTrials.gov and published articles

The total sample of RCTs (n = 152) included 143 randomly selected articles from 5 ICMJE journals, describing 146 RCTs with a unique registry identifier (NCT) in ClinicalTrials.gov and all 6 articles (6 RCTs) from 2 ICMJE journals (Figure 1). Other ICMJE journals did not publish reports of RCTs and were not included in the study. To test whether the number of published articles varied over time analyzed in the study, we compared this number for journals with more than 20 published articles in three arbitrary time periods between September 2005 and April 2008 and did not find any differences (χ^2^_2_ = 0.009, P = 0.995 for *NEJM*; χ^2^_2_ = 0.005, P = 0.997 for *JAMA*; χ^2^_2_ = 0.042, P = 0.980 for *The Lancet*; χ^2^_2_ = 2.825, P = 0.243 for *Ann Int Med*; and χ^2^_2_ = 4.755, P = 0.093 for *BMJ*).

The main characteristics of the selected RCTs from the ClinicalTrials.gov and articles are presented in Table S2. The majority of registered RCTs were phase 3 (n = 80, 52.6%), placebo controlled (n = 63, 41.4%), double blind (n = 85, 55.9%), with parallel assignment (n = 103, 67.8%) and with efficacy and safety end point (n = 75, 49.3%). The most common purpose was treatment (n = 102, 67.1%), with both genders as participants (n = 131, 86.2%). Only 9 (5.9%) RCTs were single-center, the intervention was mostly a drug (n = 100, 65.8%) and more than half (86, 56.6%) were not sponsored by industry. Finally, most RCTs (n = 96, 63.2%) included both primary and secondary outcomes and were superiority studies (n = 130, 85.5%). Positive results were reported for 77 (50.7%) RCTs, serious adverse events were published in 131 (86.2%) articles and 7 (4.6%) RCTs were reported to be stopped early for harm and 3 (2.0%) for benefit.

### Time of RCT registration in ClinicalTrials.gov vs. ICMJE deadlines

Two thirds of the RCTs (n = 101, 66.4%) were registered before 13 September 2005 deadline. In relation to the recruitment status, 118 (77.6%) started before and 31 (20.4%) after the registration. For 3 (2.0%) RCTs there were no data about the start date in the registry.

### Completeness of Minimum Data Set items in ClinicalTrials.gov

Out of 152 RCTs, 29 (19.1%) had a single registration point before the publication and 123 RCTs (80.9%) had 1 or more changes to the registered data from the first registration to the publication.

At the time of registration, 152 RCTs had a total of 224 missing registry fields for 9 WHO Minimum Data Set items (Table 1). For 101 missing registry fields (45.1% of all missing fields), the data could be found in some other field, mostly in the Detailed description field of the ClinicalTrials.gov. Most commonly missing field was ‘Key secondary outcomes’ (44.1% RCTs), followed by ‘Primary outcome’ (38.8% RCTs). For a half of the ‘Key secondary outcomes’ entries (50.7%) and for more of the three quarters of the ‘Primary outcomes’ entries (81.3%), the data were found in other fields (Table 1).

**Table 1:** Randomized controlled trials (RCTs) in ClinicalTrials.gov with missing data in the registration fields of 9 WHO Minimum Data Set items at initial registration and at last change before publication date, and differences of published data to those specified in the trial registration.

	No. (%) of RCTs with missing or misplaced registry data				
	at initial registration (n = 152)		last change before publication date (n = 123)†		
Minimum Data Set items*	missing in relevant registry field	present in other registry field	missing in relevant registry field	present in other registry field	Reported data vs registry data:No. (%) of published RCTs different from registration (n = 152)‡
10. Official scientific title of the study	26 (17.1)	0 (0.0)	14 (11.4)	0 (0.0)	111 (73.0)
12. Condition	0 (0.0)	0 (0.0)	0 (0.0)	0 (0.0)	0 (0.0)
13. Intervention(s)	3 (2.0)	3 (2.0)	2 (1.6)	1 (0.8)	113 (74.3)
14. Key inclusion or Key exclusion criteria	3 (2.0)	0 (0.0)	3 (2.4)	0 (0.0)	44 (28.9)78 (51.3)
15. Study type	0 (0.0)	0 (0.0)	0 (0.0)	0 (0.0)	21 (13.8)
16. Anticipated trial start date	22 (14.5)	1 (0.6)	8 (6.5)	1 (0.8)	34 (22.4)
17. Target sample size	44 (28.9)	18 (11.8)	36 (29.3)	13 (10.6)	118 (77.6)
19. Primary outcome	59 (38.8)	48 (31.6)	42 (34.1)	35 (28.4)	59 (38.8)
20. Key secondary outcomes	67 (44.1)	34 (22.4)	49 (39.8)	30 (24.4)	98 (64.5)

*WHO Minimum Data Set items relevant for publication and assessment of trial quality. Other 11 items were either general or administrative information, recorded only in the recruitment period and not routinely published in a journal article.

† 29 trials had a single registration entry before publication.

‡ Difference from the last change in the registration for RCTs with more than 1 registration change or difference from the first registration data for RCTs with a single registration entry (no change in the registration).

For RCTs with more than 1 change in the registration entries (n = 123), the number of missing items in relevant fields at the last registration change before publication did not significantly differ from that at the first registration (chi-square test, *P* range 0.243–0.940 for individual registration items). There were no differences in the number of RCTs registry fields missing at initial registration at the last change in the registration before publication in individual journals (Table S3). Similar to the finding at first registration, the most commonly missing fields in at the last registration were ‘Key secondary outcomes’ (39.8% RCTs) and ‘Primary outcome’ (34.1% RCTs) and the information on a more than half of those missing items could be found in other registry fields (Table 1).

Significantly more RCTs starting recruitment after registration than RCTs starting recruitment before registration had missing data about the scientific title of the study, intervention(s), key inclusion or exclusion criteria, anticipated trial start date, target sample size, primary outcome or key secondary outcomes. At initial registration, 57 (48.3%) out of 118 trials starting before and 24 (77.4%) out of 31 trials starting after registration had missing data for at least one of the registry items (χ^2^_1_ = 7.255, *P* = 0.007). At the last change in the registry before publication, 39 (42.4%) out of 92 trials starting before and 21 (72.4%) out of 29 trials starting after registration had missing items (χ^2^_1_ = 6.795, *P* = 0.009).

### Changes in Minimum Data Set items in ClinicalTrials.gov

For 123 RCTs that had recorded changes in the ClinicalTrials.gov before the publication of the article in the journal, the median number of all changes (major and minor) was 4 (95% CI 3–5, range 1 to 38). Major changes were found for 8 out of 9 WHO Minimum Data Set items (Table 2) and in 31 (25.2%) RCTs registrations: 16 out of 60 published in *N Engl J Med*, 9 out of 29 in *The Lancet*, 6 out of 33 in *JAMA* and 3 out of 12 in *Ann Intern Med*. A single change was observed for 10 RCTs, mostly concerning the ‘Target sample size’ entries. Out of 21 RCTs with more than 1 change, only in a single case the change did not include primary or secondary or both outcomes. The changes occurred mostly often for ‘Primary outcome’, ‘Key secondary outcomes’ and ‘Target sample size’ entries (Table 2). The changes in the entries for primary and secondary outcomes occurred at the same time for 19 RCTs and 8 RCTs with a change in both primary and secondary outcomes also had 1 to 3 other changes at the same time. More RCTs that registered the trial before patient recruitment underwent major changes to dataset items then those registered after the start of the patient recruitment: 20 out of 31 (64.5%) vs. 11 out of 118 (9.3%), respectively (χ^2^_1_ = 40.103, *P*<0.001).

**Table 2:** Major changes in ClinicalTrials.gov registry fields for 8 WHO Minimum Data Set items in 31 RCTs out of 123 RCTs with one or more changes in the registry after the initial registration.*

WHO Dataset item	Change	No. (%) RCTs
**Item 10 – Official scientific title of the study**	Added at last change before publication	11 (8.1)
**Item 13 – Intervention(s)**	Code changed to generic name	1 (0.8)
**Item 14 – Inclusion criteria**	One criterion deleted	1 (0.8)
**Item 14 – Exclusion criteria**	One criterion deleted	1 (0.8)
**Item 15 – Study type**	Changed from “not applicable” to phase 3	1 (0.8)
**Item 16 – Anticipated trial start date**	Added at last change before publication	7
	Changed to earlier date	1
	Total	8 (6.5)
**Item 17 – Target sample size**	Added at last change before publication	10
	Existing entry deleted	1
	Total	11 (8.9)
**Item 19 – Primary outcome**	New outcomes added at last change before publication	20
	Existing outcome deleted	1
	Total	21 (17.0)
**Item 20 – Key secondary outcomes**	New outcomes added at last change before publication	18
	Existing outcome deleted	1
	Total	19 (15.4)

*Major modification of the registered data was defined as either a qualitative change – difference in the meaning of the information provided in the registry field, or a quantitative change – difference in the numerical entry in the registry field. For 1 out of 9 evaluated items (Item 12 ‘Health Condition(s) or Problem(s) Studied’), we did not record any changes.

### Reporting completeness of registered Minimum Data Set items

The comparison of the data published in journal articles with either the data from the initial registration or the last registration change before submission to publication, revealed differences in 8 and no changes in 1 dataset item (Item 12 ‘Condition’) out of 9 analyzed dataset items (Table 1). The number of RCTs with differences between registered and published data ranged from 21 (13.8%) for ‘Study type’ to 118 (77.6%) for ‘Target sample size’ entries (Table 1). More than half of RCTs had different registry and publication data for the ‘Intervention(s)’, ‘Official scientific title of the study’, ‘Key secondary outcomes’ and ‘Key exclusion criteria’ entries. As shown in Table 3, the differences in the published data vs. registered items involved clarification of the entry in the article, especially for the registry entries ‘Official scientific title of the study’, ‘Intervention(s)’ and ‘Study type’. For some of the entries, such as ‘Key inclusion and exclusion criteria’ and ‘Primary and Secondary outcomes’, the information in the article differed considerably from the registered data. The most common difference was the addition of one or more inclusion or exclusion criteria and addition of primary or secondary outcomes that could not be found in the registry.

**Table 3:** Major changes of 8 WHO Minimum Data Set items* in published articles compared to the last ClinicalTrials.gov registry data before publication for 152 published RCTs in ICMJE journals.

ICMJE/WHO Dataset item/change	Change	No. (%) RCTs
**Item 10 – Official scientific title of the study**	More informative in article then in registry	54
	More informative in registry then in article	41
	Missing in registry	14
	Different in article from registry	2
	Total	111 (73.0)
**Item 13 – Intervention(s)**	More informative in article	110
	Missing in registry	1
	Different in article from registry	2
	Total	113 (74.3)
**Item 14 – Inclusion criteria**	New criteria added in article (median 2.0, 95% CI 1.84–2.55)	25
	Criteria omitted in article (median 2.0, 95% CI 1.96–2.76)	19
	New added and some omitted	2
	Missing in registry	1
	More informative in article then in registry	1
	Total	44 (28.9)
**Item 14 – Exclusion criteria**	New criteria added in article (median 4.0, 95% CI 3.80–6.73)	37
	Criteria omitted in article (median 3.5, 95% CI 3.34–5.54)	36
	New added and some omitted	4
	Missing in registry	9
	Total	78 (51.3)
**Item 15 – Study type**	More informative in article then in registry	19
	Different in article from registry†	2
	Total	21 (13.8)
**Item 16 – Anticipated trial start date**	Started later (according to date in article vs. in registry)	12
	Started earlier (according to date in article vs. in registry)	5
	Missing in registry or article	17
	Total	34 (22.3)
**Item 17 – Target sample size**	Greater in article then in registry	54
	Smaller in article then in registry	32
	Missing in registry or article	32
	Total	118 (77.6)
**Item 19 – Primary outcome** ‡	New outcomes introduced in article	3
	Registered outcomes omitted in article	2
	Changed to secondary outcome in article	3
	Stated in article but missing in registry	46
	Newly introduced and reported as secondary	1
	Uninformative or not reported separately from secondary outcomes	4
	Total	59 (38.8)
**Item 20 – Key secondary outcomes** †	New outcomes introduced in article	19
	Registered outcomes omitted in article	15
	Stated in article but missing in registry	48
	Outcome used for power calculation in article different from registered	2
	Timing of assessment different in article and registry	1
	Some newly introduced and some omitted at the same time	3
	Combination of newly introduced or omitted outcomes and difference in power calculation	4
	Uninformative or not reported separately	6
	Total	98 (64.5)

*For 1 out of 9 items (Item 12 ‘Health Condition(s) or Problem(s) Studied’), no changes were recorded.

† Studies registered as observational but published as RCTs.

‡ Classification of changes modified from Chen et al. [8].

At the time of analysis in 2008, we found changes in the registry fields that occurred after the publication of trial results in a journal (2 in *JAMA*, 3 in *The Lancet* and 3 in *N Eng J Med*). Out of these, 6 were sponsored by industry and all of them involved change in the registry fields for ‘Primary and/or Secondary outcomes’. In addition to the ‘Primary and Secondary outcomes’, ‘Scientific Title’, ‘Start date’ and ‘Sample size’ entries were changed in the registry for 1 published RCT. For another RCT, there was a change in ‘Sample size’ and ‘Primary and Secondary outcomes’ (both RCTs were published in *The Lancet*).

## Discussion

Our study demonstrated that the information in the ClinicalTrials.gov registry was often incomplete for the trials whose results were published in the ICMJE member journals during the evaluation period of first 2.5 years after the implementation of their trial registration policy in 2005. Incompleteness of trial registration and selective reporting of registered data has been shown for different subsets of journals and databases [5]–[8], [10]–[12], [32]–[33] but our study specifically assessed ICMJE member journals which were the first to develop and implement the registration policy. To the best of our knowledge, our study is also the first one to investigate the changes to the registration up to the publication of the trial results. Significant changes occurred in many registry entries between the initial registration and article publication, including substantive changes to the primary outcome in 17% and key secondary outcomes in 15% of the trials included in this study. The number of RCTs with missing or uninformative information at initial registration decreased at the time of the last change in the registry before the publication, but this change did not reach statistical significance. Even at the last registry change before the publication, more than a third of RCTs had missing information on primary or secondary outcomes in relevant registry fields or had this information in other registry fields. Finally, journal articles on registered trials presented information different from that in the registry for many WHO Minimum Data Set items, including key secondary outcomes (64% RCTs), target sample size (78% RCTs), interventions (74% RCTs), exclusion criteria (51% RCTs) and primary outcome (39% RCTs).

Our study assessed a single registry, ClinicalTrials.gov, which presents a study limitation. However, at the time when the ICMJE mandatory registration policy was announced and implemented, ClinicalTrials.gov was the only registry that fulfilled the ICMJE criteria [23] and the greatest increase in registered trials around ICMJE deadline dates was observed for this registry [26]. Another limitation may be that ClinicalTrials.gov does not necessarily mandate certain data items that are mandatory in any WHO primary register [31]. At present, ClinicalTrials.gov is not a member of the Network of WHO Primary registries but is a data provider to the ICTRP [31]. A further limitation of the study is its retrospective design and a small number of RCT registered after ICMJE deadline and before the recruitment of the first patients (20.4% of the whole study sample). Also, the analysis included a random sample of articles from ICMJE journals publishing many RCTs and all articles from 2 ICMJE journals with small number of published RCTs, which may have introduced a bias. The temporal comparison of registration items and changes after registration was assessed in two time periods, before and after the ICMJE policy deadlines, so that categorization of the time as a continuous variable may have introduced a bias in the statistical analyses. The categorization was used because of the evidence that the highest RCTs registration volume occurred before the September 2005 deadline [25]. The same bias may have been introduced for the analysis of changes in the registry over time because these were categorized to changes at initial registration and at last change before publication. We focused on the first two years after the registration policy implementation because this time frame was set by the ICMJE as the period for policy evaluation as a basis for possible future changes to the policy [24]. Our study period was six months longer then the initial two years because we considered the first six months after the September 2005 ICMJE deadline as an adaptation period for sponsors, researchers and editors. The trial registration landscape has been changing since the introduction of ICMJE registration requirement [32], [35] and this may have affected our results, as well as any longer follow-up of registration since the ICMJE registration deadlines in 2005. Finally, an important limitation of the study could be that the assessment of registry entries, especially the precision of information and qualitative changes in the registry fields during the trial, was inherently subjective. However, the evaluation of the registry entries and the published articles was performed by a clinical pharmacologist (MH) with experience in different types of clinical trials as well as in reporting standards for RCTs.

We found that significantly more RCTs which started recruitment after registration had missing registry data in important WHO Minimum Data Set items at the first registration and at last change before publication than RCTs which started recruitment before the registration (ie, RCTs that had to be registered until ICMJE deadline of 13 September 2005). The explanation for such finding could be that the researchers or sponsors who were responsible for the registry data entry had greater incentive for a more complete registration at the point when the trial was either well advanced or its results prepared for a publication then those who had to enter data for a future trial. This explanation is also supported by our finding that major changes to the registry entries were more frequent for RCTs registered before the recruitment then for those registered after the onset of recruitment. For trials with prospective registration, although changes to the some registered data are expected during a trial execution, major changes such as deleting or adding primary outcomes, as observed in our study, should not occur.

We found 14 RCTs that declared only a drug code in the ‘Intervention’ registry field and in only 2 of those the code was changed to a generic name at the last registry change before the publication. Clearer and more honest reporting is now promoted by the change to WHO Minimum Data Set requirement for this item, from the statement “For an unregistered drug, the generic name, chemical name, or company serial number is acceptable.” [34] to the statement “For investigational new drugs that do not yet have a generic name, a chemical name, company code or serial number may be used on a temporary basis. As soon as the generic name has been established, update the associated protocol records accordingly.” [35].

At the time of the ICMJE policy implementation, the problems of data quality in the ClinicalTrials.gov already existed. For example, Zarin et al [26] reported that out of 2670 studies registered in this registry between May and October 2005, almost a quarter failed to enter any information for Item 19 ‘Primary outcome’ and many had noninformative entries for Item 13 ‘Intervention’. Our study showed that introduction of the ICMJE policy of providing WHO Minimum Data Set did not result in better registry data, either for the trials that had to be only registered before submission to the journal or the trials that needed registering before the trial onset. The same was true for other 21 trial registries evaluated from the period from April 2005 and February 2007 [33].

It is not known whether or how ICMJE editors verified the compliance with the WHO Minimum Data Set during the period analyzed in our study. Our journal, the *Croatian Medical Journal*, is an ICMJE member journal and published 5 RCTs during the investigated period. All RCTs had to be registered to comply with the 13 September 2005 deadline and we did not check the adequacy of registration. We are not sure about the practices of journals with a high number of RCT submissions. The time around the two deadlines was surely a busy one for editors of such journals, who had to train the staff as well as train and assist their authors in implementing the registration policy. One of the reasons for poor compliance with the WHO Minimum Data Set during the analyzed period could also be the presentation of registry elements in the ClinicalTrials.gov at that time, which included a number of data fields other than the WHO Minimum Data Set. Only in September 2007, ClinicalTrials.gov introduced the tabular view of the registry items, where WHO Minimum Data Set items were clearly indicated by different color [36]. As the number of RCTs from our study registered and submitted to ICMJE journals after September 2007 was small, we could not assess if new features of ClinicalTrials.gov facilitated the editorial work and increased compliance with the WHO Minimum Data Set after 2007.

Also, it is not known whether ICMJE (and other) editors make use of the *Archive* site of the ClinicalTrials.gov to assess possible significant changes from the initial trial registration, which could influence the peer review and editorial decisions. We found that important registry data in the sample of articles and trial registrations in our study changed during the registration up to the journal publication but have no information whether these changes were addressed and resolved between the authors and editors during manuscript processing and publication. We observed that the data in the registry for several RCTs changed even after the publication of trial results and involved changes to the primary and secondary outcomes. These changes may be related to the legal requirements for trial results registration, introduced in September 2007, when Food and Drug Administration Amendments Act (FDAAA) mandated that all phase 2–4 drug and biological studies with one or more study sites in the United States, or studies being conducted under a U.S. investigational new drug application, be registered in ClinicalTrials.gov within 21 days of first patient enrolment and that trials that were in progress should be registered by December 2007 [37].

What are the implications of the study results and what can editors and other stakeholders in clinical research do to improve the quality of registry data? We believe that the quality assurance and management cannot be left only to the registries, but that the journals should continue to carry burden for the transparency of clinical research. An important step in increasing the compliance with the WHO Minimum Data Set could be that ICMJE (and other) journals clearly describe their procedures for ensuring the full implementation of their registrations requirement policy, primarily how they ensure that all relevant fields contain meaningful information, whether they assess the changes to the registration data and how they handle important discrepancies. With a powerful tool such as the *Archive* site of the ClinicalTrials.gov and other registries and a clear description of ‘sentinel’ procedures to ensure the quality of registered data, journals may provide strong incentives for authors and sponsors for better compliance with the WHO Minimum Data Set. We can envision a trial registration checklist similar to those for reporting results from different types of research, such as CONSORT [38]. Similar to the CONSORT checklist, peer reviewers could be asked for quality check with the registration checklist; the final check of the results in a submitted manuscript against the registry data would be performed by journal editors. All discrepancies between the manuscript and the registration data should be resolved before final approval for publication and addressed in the manuscript.

In addition to registries and journals, other stakeholders should contribute to the efforts to ensure that the information on clinical trials is honest, transparent and accurate. Detailed information on the WHO Minimum Data Set could be a part of the application process for trial funding and the approval of the ethics committees and regulatory authorities, which could perform a quality check of the registration data planned for a public register before providing approval for the trial. It is not clear whether researchers doing systematic reviews of trials use the *Archive* site but it may provide a powerful tool for them to check the quality of the data included in the review. Archiving of changes to the registration should in now a standard feature for all primary registries within the WHO ICTRP, where each registry has to provide an audit trail of changes to trial profiles in order to maintain primary status [31]. From 2010, the checklist of the CONSORT statement includes the need for specification of any changes to trial outcomes after the trial commenced, with reasons, as well as where the full trial protocol can be accessed, if available [38]. ClinicalTrials.gov has also recently introduced separate registry fields for original and current primary and secondary outcome measures.

Both the academic and industry researchers may also need more information about registration standards before they are expected to comply with the current requirements. A survey of academic researchers about their opinion regarding the registration of study details showed that only 31% were willing to disclose study document, particularly study protocols and financial agreements [39]. Another survey showed that 62% trial researchers with non-industry funding and 42% researchers with mixed or only industry funding would always provide the WHO Minimum Data Set to a public registry for future clinical trials [40].

Our study and the recent evaluation of the compliance of 21 trial registries with the WHO Minimum Data Set [33] show that there is a need for standardization of mandatory dataset items across the registries and in collaboration with the ICMJE. Registry items that differ or are missing from registries, such as ethics committee approval, regulatory authority approval, assessment of adverse event, funding source, should be standardized in order to improve quality and completeness of subsequent publications. For example, the WHO Minimum Data Set does not include statement on assessing adverse events (AE), although in the sample of the RCTs analyzed in our study, serious and non-serious AEs were mentioned in more than 80% of the published articles. Several studies have documented underreporting of low-grade AEs, underreporting of recurrent AEs and inconsistent and incomplete characterization and reporting of high-grade AEs [10]–[12]. Introducing entries addressing safety issues in relation to registered outcome measures could be the solution to this problem. Trial registries also contain limited methodological information such as random sequence generation, allocation concealment, sample size calculation, all important for critical appraisal of trial results [41]. These are not mandatory items of the WHO Minimum Data Set or ICMJE requirements. The WHO ICTRP has recently introduced a number of measures to improve the quality of registered data, such as improvement of explanatory text on trail registration data set and establishment of International Standards for Clinical Trial Registries [42].

In conclusion, although the introduction of ICMJE registration policy increased the visibility of clinical trials, there is a need for further improvement of quality control procedures in the journals so that they continue to lead the improvements in the transparency of clinical trials. We hope that our assessment of the first two years of the registration policy implementation would provide evidence for the ICMJE to assess the structures and procedures it has now in place so that the outcomes of the trial registration policy could be further improved.

## Supporting Information

Table S1 **Registration items in** ClinicalTrials.gov **(** CT.gov **) in relation to ICMJE requirements.**
(DOC)

Table S2 **Characteristics of RCTs included in the study.**
(DOC)

Table S3 **Missing registry data in individual journals.**
(DOC)
