# Supplementary material for: Completeness and Changes in Registered Data and Reporting Bias of Randomized Controlled Trials in ICMJE Journals after Trial Registration Policy
Source: PLoS One. 2011 Sep 21;6(9):e25258. doi: 10.1371/journal.pone.0025258 (PMC3177887; doi:10.1371/journal.pone.0025258)
Supplement: Table S1 — Registration items in ClinicalTrials.gov ( CT.gov ) in relation to ICMJE requirements. (DOC) [file pone.0025258.s001.doc]

**Table S1.** Mandatory and optional data elements for registration in *ClinicalTrials.gov* (CT.gov) in relation to WHO Minimum Data Set requirements adopted by ICMJE*

| **WHO Minimum Data Set requirements** | **CT.gov requirements†** |
| --- | --- |
| 1. Primary Registry and Trial Identifying Number | Assigned by the system |
| 2. Date of Registration in Primary Registry | Assigned by the system |
| 3. Secondary Identifying Numbers | Mandatory: CT.gov, FDAA |
| 4. Source(s) of Monetary or Material Support | Optional |
| 5. Primary Sponsor | Mandatory: CT.gov, FDAA |
| 6. Secondary Sponsor(s) | Optional |
| 7. Contact for Public Queries | Mandatory: CT.gov, FDAA |
| 8. Contact for Scientific Queries | Optional |
| 9. Public Title | Mandatory: CT.gov, FDAA |
| **10. Scientific Title** | Optional |
| 11. Countries of Recruitment‡ | Mandatory: CT.gov, FDAA |
| **12. Health Condition(s) or Problem(s) Studied** | Mandatory: CT.gov, FDAA |
| **13. Intervention(s)** | Mandatory,  Intervention Type: CT.gov, FDAA  Intervention Name: CT.gov, FDAA  Intervention Description: FDAA |
| **14. Key Inclusion and Exclusion Criteria** | Mandatory: CT.gov, FDAA |
| **15. Study Type** | Mandatory: CT.gov, FDAA |
| **16. Date of First Enrollment** | Mandatory: FDAA |
| **17. Target Sample Size** | Mandatory: FDAA |
| 18. Recruitment Status | Mandatory: CT.gov, FDAA |
| **19. Primary Outcome(s)** | Mandatory: FDAA |
| **20. Key Secondary Outcomes** | Mandatory: FDAA |

*Definitions and explanation available at <http://www.who.int/ictrp/network/trds/en/index.html>. Data items in bold were analyzed in the study.

†Definitions and explanation available at <http://prsinfo.clinicaltrials.gov/definitions.html> and <http://prsinfo.clinicaltrials.gov/who-icmje-crosswalk.html>. Some data elements were mandatory by ClinicalTrials.gov, and some were introduced in 2007 to comply with US Public Law 110-85, Section 801 (Food and Drug Administration Amendments Act of 2007, FDAA). FDAA set or requirements related to trial registration took an effect on 26 December 2007 [37].

‡In its 2005 statement [24], ICMJE defined Item 11 as ‘Positive research ethics review’; this was replaced by ‘Countries of Recruitment’ in 2006 [25].
