# Supplementary material for: Completeness and Changes in Registered Data and Reporting Bias of Randomized Controlled Trials in ICMJE Journals after Trial Registration Policy
Source: PLoS One. 2011 Sep 21;6(9):e25258. doi: 10.1371/journal.pone.0025258 (PMC3177887; doi:10.1371/journal.pone.0025258)
Supplement: Table S2 — Characteristics of RCTs included in the study. (DOC) [file pone.0025258.s002.doc]

**Table S2.** Characteristics of RCTs included in the study (n=152), according to the data from *ClinicaTrials.gov* and published articles

| **Data from *ClinicaTrials.gov*** | | | | | | |
| --- | --- | --- | --- | --- | --- | --- |
|  | | **No. RCTs (%)** | |  | | **No. RCTs (%)** |
| ***Study phase:***  2  2/3  3  4  NA*  Missing data  Inaccurate entry† | | 18 (11.8)  8 (5.3)  80 (52.6)  21 (13.8)  3 (2.0)  22 (14.5)  41 (27.0) | | ***Maximum age of participants:***  Defined (median 65.0, range 0.01-95.0)  Missing data | | 70 (46.1)  82 (53.9) |
| ***Blinding:***  Open  Single blind  Double blind  Missing data  Inaccurate entry | | 39 (25.7)  9 (5.9)  85 (55.9)  19 (12.5)  12 (7.9) | | ***Sample size:***  Defined (median 600, range 32-60000)  Missing data | | 122 (80.3)  30 (19.7) |
| ***Control:***  Placebo  Active  NA  Missing data  Inaccurate entry | | 63 (41.4)  58 (38.2)  1 (0.7)  30 (19.7)  29 (19.1) | | ***Condition:***  Heart and blood  Cancer  Behavioral and mental  Nutrition and metabolic  Digestive  Immune system  Respiratory  Blood and lymph  Bacterial and fungal  Urinary and sex and pregnancy  Muscle and bone  Viral  Other | | 36 (23.7)  16 (10.5)  9 (5.9)  9 (5.9)  8 (5.3)  7 (4.6)  7 (4.6)  7 (4.6)  6 (3.9)  6 (3.9)  6 (3.9)  6 (3.9)  29 (19.0) |
| ***Assignment:***  Parallel  Factorial  Single group  Missing data  Inaccurate entry | | 103 (67.8)  8 (5.3)  10 (6.6)  31 (20.4)  42 (27.6) | | ***Intervention type:***  Drug  Procedure and surgery  Behavioral  Device  Biological and vaccine  Drug + Procedure and surgery  Other  Missing data  Inaccurate entry | | 100 (65.8)  12 (7.9)  10 (6.6)  7 (4.6)  7 (4.6)  3 (2.0)  7 (4.6)  6 (5.8)  14 (9.2) |
| ***Endpoint:***  Safety  Efficacy  Efficacy and safety  Accuracy  Missing data  Inaccurate entry | | 5 (3.3)  37 (24.3)  75 (49.3)  1 (0.7)  34 (22.4)  39 (25.7) | | ***Intervention name:***  Specific  Nonspecific  Missing data  Inaccurate entry | | 136 (89.5)  15 (9.9)  1 (0.7)  16 (10.6) |
| ***Purpose:***  Treatment  Prevention  Diagnostic  Screening  Other  Missing data  Inaccurate entry | | 102 (67.1)  34 (22.4)  1 (0.7)  1 (0.7)  5 (3.4)  9 (5.9)  15 (9.9) | | ***Sponsor:***  Non industry  Industry  Joint sponsorship | | 86 (56.6)  61 (40.1)  5 (3.3) |
| ***Recruitment:***  Completed  Other  Missing data  Inaccurate entry | | 117 (77.0)  34 (22.4)  1 (0.7)  29 (19.1) | | ***Outcome measures:***  Primary outcome  Primary and secondary outcomes  Missing data:  - data visible elsewhere in register | | 13 (8.6)  96 (63.2)  43 (28.3)  37 (24.3) |
| ***Center:***  Multicenter  Single center  Inaccurate entry | | 55 (36.2)  9 (5.9)  6 | | ***Primary outcome:***  Defined  Clinical  Surrogate | | 117 (77.0)  88 (57.9)  39 (25.7) |
| ***Participants’ gender:***  Both  Female  Male  Missing data | | 131 (86.2)  17 (11.2)  3 (2.0)  1 (0.7) | | ***Secondary outcomes:***  Defined  Clinical  Surrogate | | 100 (65.8)  78 (51.3)  69 (45.4) |
| ***Minimum age of participants:***  Defined (median 18.0, range 0.01-60.0)  Missing data | | 135 (88.8)  17 (11.2) | |  | |  |
| **Data from published articles:** | | | | | | |
|  | **No. RCTs (%)** | |  | | **No. RCTs (%)** | |
| ***Funding:***  Identical as sponsor  Not identical as sponsor  Partially identical‡  Missing data | 105 (69.1)  14 (9.2)  29 (19.1)  4 (2.6) | | ***Adverse events – Nonserious:***  Yes  No  Missing data | | 126 (82.9)  25 (16.4)  1 (0.7) | |
| ***Ethics committee approval:***  Yes  No | 151 (99.3)  1 (0.7) | | ***Trial aim:***  Superiority  Noninferiority  Equivalence  Noninferiority+ Superiority  Missing data | | 130 (85.5)  11 (7.2)  8 (5.3)  2 (1.3)  1 (0.7) | |
| ***Adverse events – Serious:***  Yes  No  Missing data | 131 (86.2)  19 (12.5)  2 (1.3) | | ***Outcome:***  Positive results  Negative results  Both positive and negative results | | 77 (50.7)  39 (25.7)  36 (23.6) | |
| ***Duration of the study:***  Same as data in registry  Stopped early for harm  Stopped early because of Safety Monitoring Committee§  Stopped early for harm + Safety Monitoring Committee  Stopped early for benefit  Longer duration than data in registry  Shorter than data in registry (follow up was shorter)  No data in registry  No data in article  Different data in summary, methods and results of the article | 105 (69.1)  7 (4.6)  4 (2.6)  1 (0.7)  3 (2.0)  2 (1.3)  1 (0.7)  2 (1.3)  1 (0.7)  25 (16.4)  3 (2.0)  1 (0.7) | |  | |  | |

*NA – not applicable.

† Inaccurate entry was defined as having unclear information or information in contradiction to the other, mostly numerical, entries in the registry. Examples include incorrect statements about the trial, such as study phase, assignment, blinding and control group, as well as study end point, purpose and recruitment status; unclear number of trial centers; and no information about the age of study participants.

‡Other sources of funding in addition to the sponsor.

§Little chance of reaching the efficacy end point by completing the trial (enrolling additional participants would not change the findings).
