# Supplementary material for: Completeness and Changes in Registered Data and Reporting Bias of Randomized Controlled Trials in ICMJE Journals after Trial Registration Policy
Source: PLoS One. 2011 Sep 21;6(9):e25258. doi: 10.1371/journal.pone.0025258 (PMC3177887; doi:10.1371/journal.pone.0025258)
Supplement: Table S3 — Missing registry data in individual journals. (DOC) [file pone.0025258.s003.doc]

**Table S3.** Randomized controlled trials (RCTs) published in individual ICMJE journals with at least 1 missing out of 9 relevant WHO Minimum Data Set registry fields at initial registration (n=152 RCTs) and at the last change in the registration before publication (n=123)

| **Journal (published RCTs with initial registration/last change before publication)** | **RCTs with missing registry data/total RCTs** | | **Statistics** |
| --- | --- | --- | --- |
|  | **at initial registration (total RCTs)** | **at last change before publication (total RCTs)** |  |
| *Ann Int Med* | 7/12 | 4/11 | χ^2^_1_=0.404, *P*=0.5240 |
| *BMJ* | 7/12 | 4/8 | χ^2^_1_= 0.008, *P*= 0.9269 |
| *Croat Med J* | 4/5 | 0/0 | – |
| *JAMA* | 20/33 | 15/29 | χ^2^_1_=0.200, *P*=0.6548 |
| *Lancet* | 13/29 | 9/19 | χ^2^_1_=0.015, *P*=0.9018 |
| *N Eng J Med* | 38/60 | 34/55 | χ^2^_1_=0.001, *P*=0.9799 |
